# Editorial: Special Issue—Understanding and Targeting Heart Failure: From Biology to Therapeutics

**DOI:** 10.3390/biology12111384

**Published:** 2023-10-30

**Authors:** Jun Pu, Wuqiang Zhu, Lei Ye

**Affiliations:** 1 Department of Cardiology, Ren Ji Hospital, School of Medicine, Shanghai Jiao Tong University, Shanghai 200127, China; pujun310@hotmail.com; 2 Department of Cardiovascular Diseases, Physiology and Biomedical Engineering, Center of Regenerative Medicine, Mayo Clinic Arizona, Scottsdale, AZ 85259, USA; zhu.wuqiang@mayo.edu; 3 Department of Biomedical Engineering, University of Alabama at Birmingham, Birmingham, AL 35233, USA

An estimated 64.3 million people are living with heart failure worldwide. In developed countries, the prevalence of known heart failure is generally estimated at 1% to 2% of the general adult population. Because of a growing and ageing population, the total number of heart failure patients still continues to rise. In recent years, the striking development of molecular and cellular biology, especially genetics, stem cell biology, and developmental biology are transforming the way we understand and treat heart failure.

In this Special Issue, entitled “Understanding and Targeting Heart Failure: From Biology to Therapeutics”, nine papers (four reviews and five research articles) which highlight recent advances in understanding the transcriptome profile in various cardiovascular diseases and providing novel treatments for promoting cardiomyocyte proliferation and cardiac repair. Reviews also explored the molecular and cellular mechanisms in the development of heart failure and the association between heart failure and cognitive impairment. 

Shen et al. [1] employed single-nuclei RNA sequencing (snRNA-seq) to evaluate the transcriptomic profile of endothelial cells (ECs) from skeletal muscles in Duchenne muscular dystrophy (DMD) mutant mice. The study showed that unique phenotypes in ECs, including suppressed expression of SPTBN1 and the upregulated expression of multiple long noncoding RNAs (lncRNAs), altered metabolic activity. Single cell RNA sequencing, which detects the mRNA level of individual cells, is a powerful tool to identify unique cell types with special gene signatures and potential signaling pathways. The downregulated expression of SPTBN1 and the upregulated expression of multiple long noncoding RNAs (lncRNAs) may lead to the altered metabolic activity in the unique ECs identified in DMD disease. 

Pulido et al. [2] analyzed transcriptome profile of ischemic and remote zones from porcine myocardial tissue. After validation with proteomics, they found that mitochondrion-related biological processes were the most impaired in the infarcted area and immune system process-related genes were upregulated in the remote tissue which was due to the increase in neutrophil migration in remote zones. This study interestingly showed increased neutrophils in remote zones of infarct, indicating that inflammation is not limited to the infarct zone, but also participates in non-infarcted myocardium in remote zone. This needs further investigation to understand the role of increased inflammation in remote zones after myocardial infarction. 

Wang et al. [3] reported the effectiveness of manganese porphyrin compound for the rescue of cardiac arrests in rodents. They showed that manganese porphyrin improved survival, neurologic function recovery, and behavioral performance in animals suffering from cardiac arrest. Ullah et al. [4] reviewed the current understanding of HIF2α and ARNT signaling in endothelial cells, their roles in inflammation and maintaining blood vessel integrity, and their involvement in ischemic heart disease. The two studies either provided new therapeutics for cardiac arrest or systematically reviewed the role of the HIF2α and ARNT signaling pathway in endothelial cells, which are helpful for establishing new therapeutics and targets for the development of new treatments for heart failure.

Strategies to induce cardiomyocyte proliferation to restore heart function after injury are being extensively explored and methods which can effectively induce the proliferation of adult mammalian cardiomyocytes are needed. Khosravi et al. [5] investigate the potential of fibroblast growth factor 10 (FGF10) and cardiotrophin-1 (CT-1) on cardiogenesis in vitro. A synergy between FGF10/CT-1 led to significantly upregulated proliferation markers, such as Aurora B Kinase and YAP, which has potential to induce mammalian cardiomyocytes re-enter the cell cycle. Bishop et al. [6] systematically reviewed and summarized the structural and functional changes of cardiomyocyte growth from hyperplasia to hypertrophy during physiological and pathological responses. As he pointed out, although evolving technology over the past century has provided an increased understanding of structural, biochemical, and functional features of the heart which have led to a better understanding of CM growth, why CM exits the cell cycle is yet to be fully understood.

Large animal models, such as the non-human primates (NHPs), are essential for translational cardiovascular research as they more closely resemble human anatomy, physiology, function, and metabolism. However, detailed characterization of NHP cardiac function is limited. Klösener et al. characterized the cardiovascular function of the common marmoset using intracardiac pressure–volume loops (PV loop) system, magnetic resonance imaging (MRI), and echocardiography [7]. This study provides the basic cardiovascular function to establish the common marmoset as a promising primate model for studying cardiovascular disease.

It is known that patients with heart failure have reduced cognition and increased dementia risk. Goh et al. [8] reviewed and discussed potential causes linking heart failure and cognitive impairment, and discussed the recognition and management of cognitive impairment in patients with heart failure.

We would like to thank all the authors for their submissions to this Special Issue. We also thank all the reviewers for dedicating their time and helping ensure the quality of the submitted papers, and, last but not least, the staff at the editorial office of *Biology* for their efficient assistance.
